# Supplementary material for: Influence of Mask Wearing during COVID-19 Surge and Non-Surge Time Periods in Two K-12 Public School Districts in Georgia, USA
Source: Int J Environ Res Public Health. 2023 May 4;20(9):5715. doi: 10.3390/ijerph20095715 (PMC10177845; doi:10.3390/ijerph20095715)
Supplement: Supplementary file 1 [file ijerph-20-05715-s001.zip › ijerph-2284502-supplementary.pdf]

Supplementary Material

Table S1. COVID-19 Rate Ratio Surge Period (August 2021) to Non-Surge Period (October 2021) for School Districts by School Level.

School District	School Level	Rate Ratio	95% Confidence Interval (LL, UL) *	p-value
Mask Optional (Cobb County School District)	Elementary School	2.89	(2.52, 3.29)	<0.001
	Middle School	3.55	(2.80, 4.54)	<0.001
	High School	3.35	(2.86, 3.94)	<0.001
Mask Required (DeKalb County School District)	Elementary School	2.03	(1.77, 2.33)	<0.001
	Middle School	2.17	(1.70, 2.77)	<0.001
	High School	2.25	(1.91, 2.67)	<0.001

* LL = lower limit, UL = upper limit.

Table S2. Community Transmission Rates During August 2021 and October 2021.

	Mask Required DeKalb County (Number of cases per 100,000)	Mask Required DeKalb County (Number of cases per 100,000)
8/7/2021 - 8/13/2021	403	414
8/14/2021 - 8/27/2021	604	505
10/2/2021 - 10/15/2021	224	287
10/16/2021 - 10/29/2021	138	115

Source: Georgia Department of Public Health County Indcator Reports (acessed 4/12/2023) [34].
